# Positron emission tomography imaging of cardiomyocyte apoptosis with a novel molecule probe [^18^F]FP-DPAZn2

**DOI:** 10.18632/oncotarget.5769

**Published:** 2015-09-21

**Authors:** Ting Sun, Ganghua Tang, Hua Tian, Kongzhen Hu, Shaobo Yao, Yifan Su, Changqian Wang

**Affiliations:** ^1^ Department of Cardiology, Shanghai Ninth People's Hospital, Shanghai JiaoTong University School of Medicine, Shanghai, P.R. China; ^2^ Department of Nuclear Medicine, The First Affiliated Hospital, Sun Yat-Sen University, Guangzhou, P.R. China; ^3^ State Key Laboratory of Oncogenes and Related Genes, Shanghai Cancer Institute and Cancer Institute of Shanghai Jiao Tong University, Shanghai, P.R. China

**Keywords:** radiosynthesis, Zinc(II)-dipicolylamine, apoptosis, cardiomyocyte, positron emission tomography imaging, Pathology Section

## Abstract

Cardiomyocyte apoptosis plays a causal role in the development and progression of heart failure. Currently, there is no effective imaging agent that can be used to detect cardiomyocyte apoptosis *in vivo*. To target phosphatidylserine (PS) on the surface of the dying cell, we synthesized a novel 18F-labeled Zn2+-dipicolylamine (DPA) analog, [^18^F]FP-DPAZn2, and evaluated it for noninvasive imaging of cardiomyocyte apoptosis. *In vitro*, the fluorescence imaging of dansyl-DPAZn2 was suitable for detecting cardiomyocyte apoptosis, which was confirmed by confocal immunofluorescence imaging, terminal dUTP nick-end labeling (TUNEL) assay, and western blot assay. The *in vivo* biodistribution showed that the uptake ratios of [^18^F]FP-DPAZn2 in the heart were 4.41±0.29% ID/g at 5 min, 2.40 ± 0.43% ID/g at 30 min, 1.63 ± 0.26% ID/g at 60 min, and 1.43% ± 0.07 ID/g at 120 min post-injection. *In vivo*, the [^18^F]FP-DPAZn2 PET images showed more cardiac accumulation of radioactivity 60 min post-injection in acute myocardial infarction (AMI) rats than in normal rats, which was consistent with the findings of a histological analysis of the rat cardiac tissues *in vitro*. [^18^F]FP-DPAZn2 PET imaging has the capability for myocardial apoptosis detection, but the method will require improved myocardial uptake for the noninvasive evaluation of cardiomyocyte apoptosis in clinical settings.

## INTRODUCTION

Myocardial cell apoptosis has been observed in various cardiovascular diseases, and it may be a key factor in a deleterious shift from cardiac compensation to decompensation [1–3]. Because the damage that results from myocardial cell apoptosis is reversible, the inhibition of myocardial apoptosis has become an effective treatment for heart failure [4, 5]. The noninvasive imaging of myocardial apoptosis could not only determine the position of this damage but also contribute to determining who would benefit from anti-apoptosis treatment and the therapeutic effects of this treatment.

*In vitro*, many methods have been successfully used for the detection of apoptosis, such as terminal deoxynucleotidyl transferase dUTP nick end labeling (TUNEL) [6], electron microscopy [7], DNA gel electrophoresis [8], and flow cytometry [9]. With the development of molecular imaging techniques, especially the clinical application of SPCET and PET, the *in vivo* noninvasive monitoring of apoptosis has become feasible. The intricate scheme of the apoptotic machinery offers several potential targets for molecular imaging. In 1992, Valerie Fadok and co-workers reported that phosphatidylserine (PS) becomes exposed on the surface of apoptotic cells by activated scramblase and inhibits aminophospholipid translocase during early apoptosis [10]. The appearance of PS on a cell surface is a general indicator of apoptosis. Therefore, surface-expressed PS provides an attractive target for the molecular imaging of apoptosis.

The protein annexin A5, which binds with high selectivity and affinity to the PS abnormally expressed on the cell membranes of apoptotic cells, is widely used for detecting apoptosis [11–13]. A number of labeled annexin A5 proteins became the first generation of tracers for the non-invasive detection of apoptosis. Labeled annexin A5 has been used in detecting apoptosis in various tumor cells; however, it is insufficient to achieve a detectable target-to-background ratio when used in cardiovascular diseases [14] because annexin A5 is a large protein with limitations, including a suboptimal biodistribution and unfavorable pharmacokinetics [15]. In 2005, Smith BD. et al [16] discovered an effective small-molecule mimic of annexin A5, a fluorescent Zn^2+^-2,2′-dipicolylamine (Zn^2+^-DPA) coordination complex, that was used to detect apoptotic cells and is superior to annexin A5 *in vitro*.

Our groups have synthesized a series of ^18^F-labeled Zn^2+^-DPA compounds used to detect apoptosis for PET imaging in living animals. PET imaging with [^18^F]FEN-DPAZn2 in normal rats shows a high uptake in the renal excretory system and sufficient clearance from most other internal organs within 1 h [17]. We have also investigated the use of [^18^F]FB-DPAZn2 in imaging apoptosis in animal models, and have found that the [^18^F]FB-DPAZn2 probe is useful for imaging ADM-treated tumor-bearing mice [18]. However, to date, the *in vivo* imaging of myocardial apoptosis using ^18^F-labeled Zn^2+^-DPA probes targeting PS has not been reported. In addition, [^18^F]FEN-DPAZn2 has a low uncorrected radiochemical yield, and 4-nitrophenyl-2-[^18^F]ﬂuoropropionate ([^18^F]NFP) is more suitable for labeling small-molecule compounds than N-succinimidyl-4-[^18^F]ﬂuorobenzoate ([^18^F]SFB) [19]. In this study, we report the PET imaging of cardiomyocyte apoptosis with a novel DPA probe, [^18^F]FP-DPAZn2.

## RESULTS

### Radiochemistry

**Figure 1:**
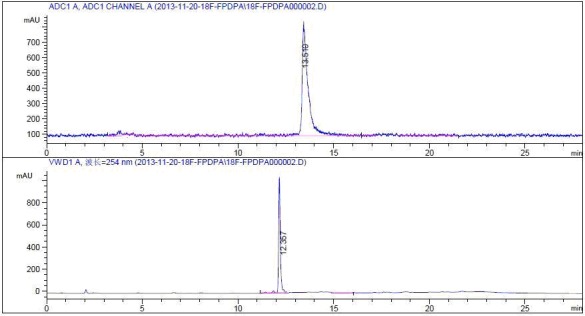
Typical HPLC-UV chromatograph (bottom) and HPLC-radioactivity detector chromatograph (top) of [^18^F]FP-DPAZn2 solution after purification

[^18^F]NFP was synthesized using the modified PET-MF-2V-IT-I synthesis module. The decay-corrected yield of [^18^F]NFP was 35 ± 5% (*n* = 10) based on [^18^F]ﬂuoride, and the synthesis time was 80 min. The decay-corrected radiochemical yield of [^18^F]FP-DPAZn2 was 90 ± 5% (*n* = 10) from [^18^F]NFP for 30 min. The total decay-corrected radiochemical yield of [^18^F]FP-DPAZn2 was 30 ± 10% (*n* = 10) from ^18^F^−^ for 110 min. The radiochemical purity of [^18^F]FP-DPAZn2 was more than 95%, and the specific radioactivity of [^18^F]FP-DPAZn2 was over 4.0 GBq/μmol. (Figure 1).

### Animal models and echocardiography

The general appearances of all of the animal groups were recorded during the time course of the study. The animals in the AMI group appeared to be sicker, weaker, and more lethargic compared with the control group. 80% of the rats on MI (8/10) survived the 24-h follow-up period, which was significantly lower than the survival rate (100%) of the rats in the control group. The AMI rats had larger heart cavities, thinner IVS and lower LVEF than normal rats when assessed by echocardiography (Table 1).

**Table 1:** Cardiac function parameters assessed by echocardiography

Characteristic	MI(n=8)	Control(n=8)	*P*
Anatomy			
BW(g)	202±10	214±8	NS
HW/BW	3.43±0.30	3.31±0.26	NS
Echocardiography			
LVEDD(mm)	7.22±0.48	6.45±0.36	0.024
IVS(mm)	1.88±0.07	2.21±0.04	0.005
LVPW(mm)	2.25±0.05	2.23±0.04	NS
LVEF(%)	47.33±9.58	82.27±4.72	0.000
HR(beats/min)	395±68	381±55	NS

NS (not significant)

### Bio-distribution studies

The bio-distribution of [^18^F]FP-DPAZn2 was evaluated in normal rats. As shown in Table 2, the highest uptake of [^18^F]FP-DPAZn2 was observed in the kidneys, followed by the liver, the pancreas, the lung, and the blood at 5-60 min post-injection; the lowest uptake of [^18^F]FP-DPAZn2 was observed in the brain, followed by the muscle, bone and stomach. The accumulation of [^18^F]FP-DPAZn2 radioactivity in the kidney was very high at early time points and decreased from 20.99 to 7.78 % ID/g with elapsing time. There was also a high accumulation of [^18^F]FP-DPAZn2 radioactivity and rapid clearance in the liver. The uptake ratios of [^18^F]FP-DPAZn2 in heart were 4.41±0.29% ID/g at 5 min, 2.40±0.43% ID/g at 30 min, 1.63 ± 0.26% ID/g at 60 min, and 1.43 ± 0.07% ID/g at 120 min post-injection. These results revealed that [^18^F]FP-DPAZn2 had a faster clearance from the kidney and a lower uptake in most tissues.

**Table 2:** Biodistribution of [^18^F]FP-DPAZn2 in normal rats (% ID/g, n = 5)

Tissue	5 min	30 min	60 min	120 min
Blood	6.30 ± 0.93	2.80 ± 0.30	2.19 ± 0.61	2.07 ± 0.19
Brain	0.72 ± 0.01	1.12 ± 0.18	0.90 ± 0.02	1.13 ± 0.20
Heart	4.41 ± 0.29	2.40 ± 0.43	1.63 ± 0.26	1.43 ± 0.07
Lung	6.24 ± 0.37	3.08 ± 0.49	2.36 ± 0.49	1.20 ± 0.14
Liver	10.97 ± 1.81	4.92 ± 0.57	4.01 ± 0.99	2.63 ± 0.35
Pancreas	7.29 ± 1.32	5.20 ± 0.97	4.08 ± 0.90	3.7 ± 0.26
Kidney	20.99 ± 5.77	12.24 ± 1.75	9.30 ± 1.61	7.78 ± 0.71
Spleen	4.03 ± 0.48	2.45 ± 0.76	1.49 ± 0.20	1.34 ± 0.35
Intestine	4.00 ± 0.73	3.59 ± 1.51	3.40 ± 0.97	3.16 ± 0.85
Muscle	1.95 ± 0.25	1.60 ± 0.11	1.31 ± 0.40	1.37 ± 0.16
Stomach	2.88 ± 0.35	2.34 ± 0.59	1.70 ± 0.33	1.32 ± 0.26
Bone	2.40 ± 0.25	1.87 ± 0.50	1.06 ± 0.38	0.25 ± 0.08

### PET imaging

**Figure 2:**
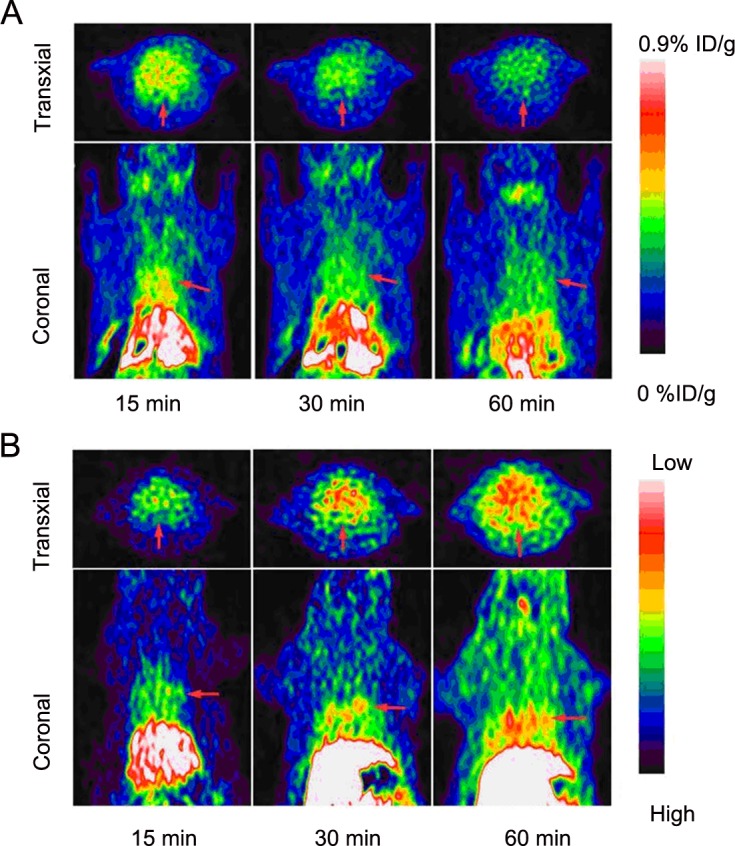
Decay-corrected transaxial and coronal [^18^F]FP-DPAZn2 PET images of the normal rats (A) and the AMI rats (B) The images were acquired 15, 30, 60 min after the injection of [^18^F]FP-DPAZn2. Short lines indicate the location of the heart.

**Figure 3:**
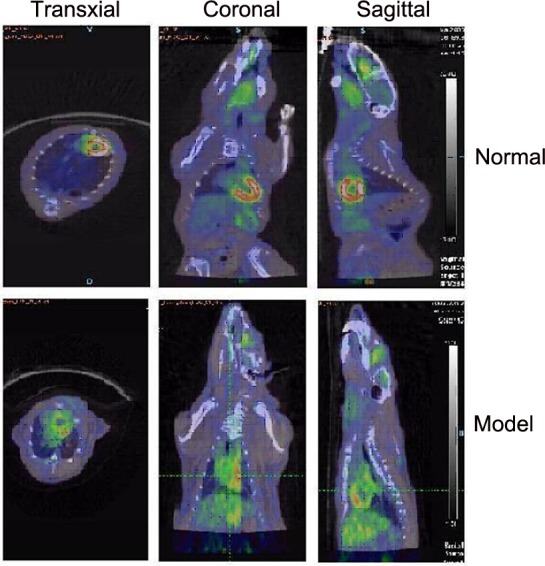
Decay-corrected transaxial and coronal [^18^F]-FDG PET/CT images of the normal rats (top) and the AMI rats (bottom) The images were acquired 60 min after the injection of [^18^F]-FDG.

Transaxial and coronal PET images of [^18^F]FP-DPAZn2 obtained in normal rats are shown in Figure 2A. The myocardial uptake of [^18^F]FP-DPAZn2 was observed at 15 min post-injection, a decreased accumulation was observed at 30 min, and there was almost no uptake at 60 min post-injection. The transaxial and coronal PET images of [^18^F]FP-DPAZn2 obtained in AMI model rats are shown in Figure 2B. There was almost no uptake of [^18^F]FP-DPAZn2 in the myocardial tissue at 15 min post-injection, an increased accumulation at 30 min, and a high uptake at 60 min post-injection. PET imaging using [^18^F]-FDG was also performed in AMI and normal rats. The decay-corrected transaxial and coronal PET images at 60 min post-injection are shown in Figure 3. The PET images show that the myocardial uptake of [^18^F]-FDG in the AMI rats was obviously decreased compared with the uptake in normal rats. Both [^18^F]FP-DPAZn2 and [^18^F]-FDG clearly revealed accumulation in the myocardial tissue. Consistently with those with [^18^F]-FDG, the [^18^F]FP-DPAZn2 PET images demonstrated that heart had the highest uptake at 60 min post-injection. However, there was a decreased [^18^F]-FDG uptake and an increased [^18^F]FP-DPAZn2 uptake in the infarcted myocardium. The ratios of accumulation in the heart to the muscle are shown in Table 3. [^18^F]FP-DPAZn2 displayed the highest heart-to-muscle uptake ratio at 60 min post-injection in AMI model rats, and the uptake ratios in the AMI model rats were higher than those in the normal rats. The ratios of accumulation of [^18^F]FP-DPAZn2 in the heart to the muscle were not significantly different between AMI rats and normal rats at 30 min post-injection.

**Table 3:** The uptake ratios of [^18^F]FP-DPAZn2 in heart to muscle (% ID/g, n = 8)

Group	Tissue	15min	30 min	60 min
AMI	Heart	0.43± 0.03	0.60± 0.05	0.68± 0.05
	Muscle	0.19± 0.02	0.20± 0.02	0.21± 0.02
	H/M ratio	2.31	2.98	3.24
Normal	Heart	0.47± 0.04	0.40± 0.04	0.35± 0.02
	Muscle	0.14± 0.02	0.15± 0.02	0.13± 0.01
	H/M ratio	3.34	2.71	2.78*

Student's t test for the heart to muscle uptake ratios of [^18^F]FP-DPAZn2 at 60 min post-injection: * *P*<0.05

### Cardiomyocyte apoptosis in histological analysis

**Figure 4:**
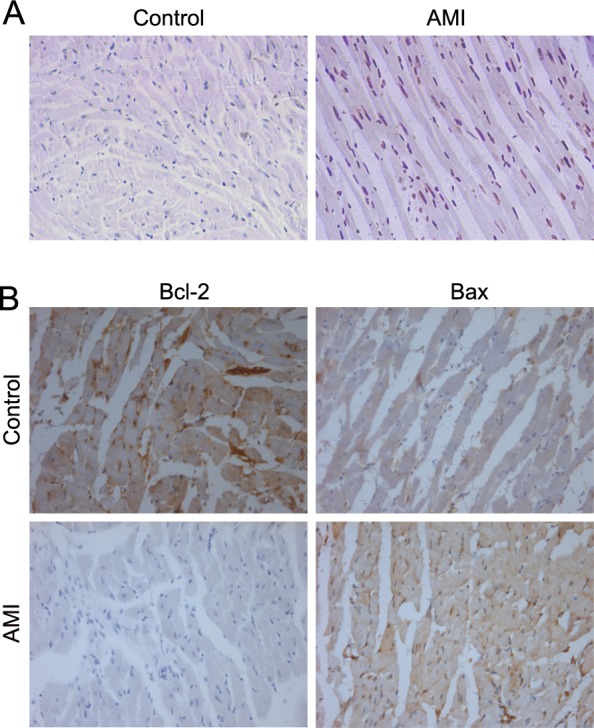
Histologic analysis of heart tissues **A.** TUNEL staining of control and heart failure tissues. **B.** Immunohistochemistry analysis of Bcl-2 and Bax in the control group and the heart failure group. Representative images are shown.

The TUNEL assay is a method for detecting DNA fragmentation by labeling the terminal end of nucleic acids. Therefore, we also detected apoptosis by the TUNEL assay. In the hearts of the control mice, TUNEL-positive cells were seldom detectable, but in heart-failure mouse hearts, numerous TUNEL-positive cells were observed (Figure 4A). The myocardial apoptotic percentages in the AMI group (27.64±3.15%) were significantly higher than those in the control group (0.57±0.21%) (*P* < 0.05). This result is consistent with the result obtained from PET imaging. Immunohistochemical staining for Bcl-2 and Bax, which is another important regulator of apoptotic cells, showed that increased Bax expression was accompanied by decreased Bcl-2 expression in the AMI group (Figure 4B). Therefore, all of these results suggest that apoptosis occurred in the myocardia of the heart-failure mice.

### Cardiomyocyte apoptosis: *in vitro* cell analysis

**Figure 5:**
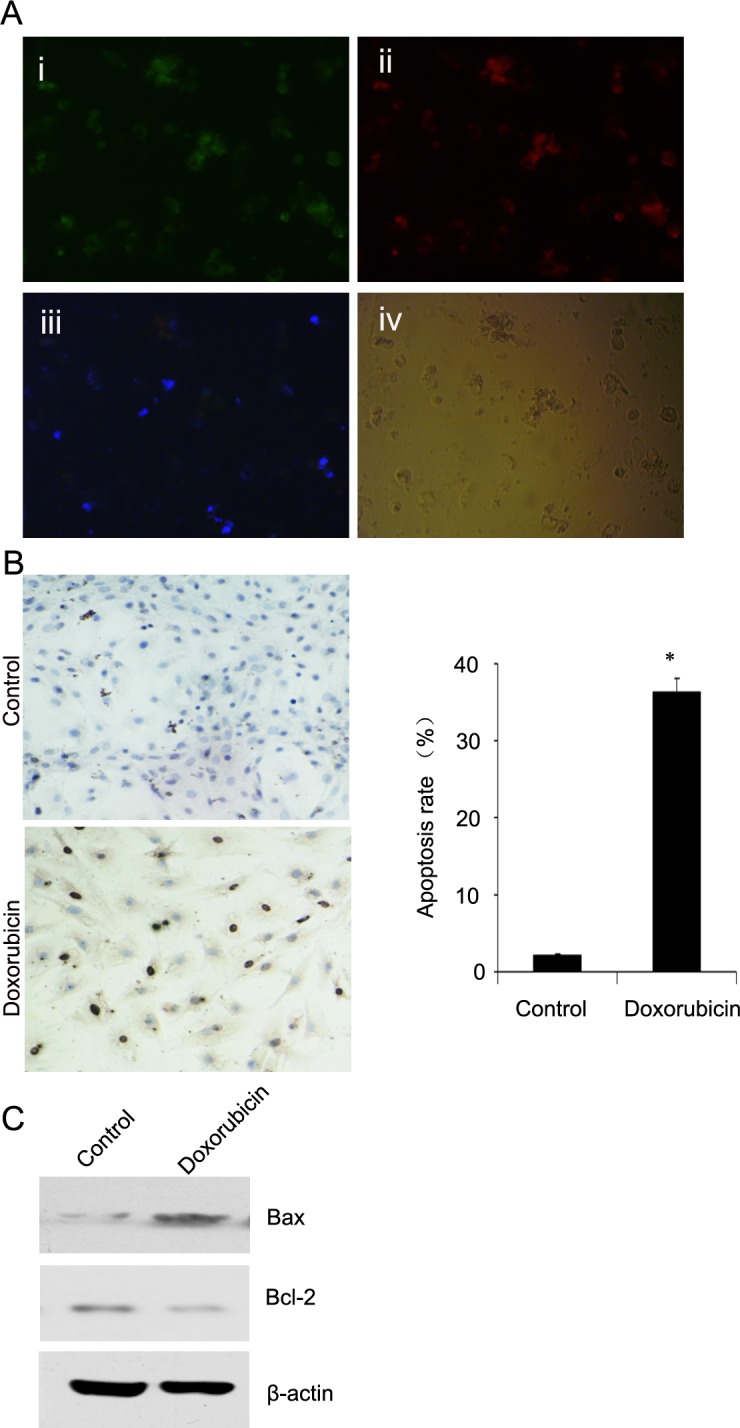
Representative images of immunofluorescent confocal imaging, TUNEL, Bcl-2 and Bax of Dox-treated H9C2 cells **A.** Morphological changes of Dox-treated H9C2 cells triply stained with FITC-Annexin V (i), PI (ii), dansyl-DPA2 (iii) and phase contrast microscopy (iv). **B.** Morphological changes of Dox-treated H9C2 cells stained with the TUNEL assay. Representative images are shown (left panel). A statistical analysis of the apoptosis rate is shown (right panel). **C.** Western blot analysis of Bcl-2 and Bax in the control group and the heart failure group. Representative images are shown.

Annexin V-FITC/propidium iodide (PI) staining was used to evaluate apoptosis as a standard control. The cells that were stained by only annexin V-FITC were defined as apoptotic. Cells that were stained by PI were defined as necrotic cells. We found that the fluorescence signal from dansyl-DPAZn2 overlapped with that from FITC-annexin V in H9C2 cells (Figure 5A). We also detected apoptosis by the TUNEL assay and a western blot assay. The results showed that numerous TUNEL-positive cells were observed in H9C2 cells treated with Dox (Figure 5B). The myocardial apoptotic percentage in the AMI group (27.64±3.15%) was significantly higher than in the control group (0.57±0.21%) (*P* < 0.05). Moreover, an increased Bax expression was accompanied by a decreased Bcl-2 expression in H9C2 cells treated with Dox (Figure 5C). Therefore, all of the results suggested that DPAZn2 may be an effective probe to detect myocardial cell apoptosis.

## DISCUSSION

Myocardial apoptosis often presents in end-stage heart failure [20] and in post-infarction LV remodeling [21–23], thus causing progressive cell loss, LV dilatation and LV dysfunction. Several studies have demonstrated that apoptosis can be manipulated and reversed [21, 24]. Early identification of apoptosis can avoid the potential loss of functional tissue and improve heart function. Thus, it is essential to pursue new noninvasive strategies for imaging myocardial apoptosis. We have designed a new probe, 2-[^18^F]fluoropropionyl-bis(zinc(II)-dipicolylamine), also called [^18^F]FP-DPAZn2, as a possible PET tracer for imaging myocardial apoptosis *in vivo*.

Apoptosis is characterized by the activation of an intracellular self-controlled and ordered energy-dependent suicide program [25]. The morphological changes associated with apoptosis include cell shrinkage, chromatin condensation and margination, expression of phosphatidylserine (PS) on the outer leaflet of the lipid bilayer, nuclear pyknosis and fragmentation, and the formation of apoptotic bodies [26]. A common technique for detecting apoptosis *in vitro* is terminal deoxynucleotidyl transferase (TdT)-mediated dUDP nick end labeling (TUNEL) staining [6], based on identifying DNA fragmentation. However, this method has limitations, including its *in vitro* and time-consuming nature, as well as the relatively late stage at which the apoptotic process is detected. Because the externalization of PS occurs early in the execution phase of apoptosis, before DNA degradation, it has become an attractive target for *in vivo* non-invasive radiopharmaceutical imaging. DPAZn2 is a convenient and relatively cheap source of PS-targeting ligands for apoptosis imaging in the clinical setting compared to other tracers. It has been reported that fluorescent Zn2+-dipicolylamine (DPA) coordination complexes have a selective affinity for membrane surfaces that are enriched in anionic phosphatidylserine (PS) and that they can be used to detect apoptosis. Therefore, we developed DPAZn2 labeled with radionuclides to better quantify apoptotic cells and improve existing DPAZn2 imaging in the cardiovascular system.

Dansyl-DPAZn2 was prepared by coupling the precursor DPA with dansyl chloride and coordinating with zinc ions. Our previous study has also shown that dansyl-DPAZn2 stains the same cells (apoptotic and necrotic cells) as fluorescein isothiocyanate (FITC) labeled annexin V (FITC-annexin V) in HeLa cells. Therefore, we also studied the effect of dansyl-DPAZn2 on cardiac myocyte apoptosis *in vitro*. The H9C2 rat cardiomyoblast cell line, an animal-free alternative, can accurately mimic the apoptotic responses of primary cardiac myocytes. Fluorescence microscopy results showed that dansyl-DPAZn2 retained the same binding activities to apoptotic H9C2 cells as those in the TUNEL assay, which meant that dansyl-DPAZn2 and TUNEL had similar abilities to detect apoptosis. Compared with TUNEL, this binding assay provides a relatively quick and convenient method to evaluate apoptosis *in vitro*. It takes only 2-3 h to complete the experiment using this method, which makes it more favorable for routine use. In the inhibited binding experiments, the dansyl-DPAZn2 stain exhibited significantly lower fluorescence staining of apoptosis than the PI double stain as the control, which demonstrated that dansyl-DPAZn2 also had the ability to selectively target apoptotic H9C2 cells with exposed anionic PS *in vitro*. These findings showed that dansyl-DPAZn2 could be a potential fluorescently labeled probe for the detection of cardiomyocyte apoptosis.

Dansyl-DPAZn2 was able to detected apoptotic H9C2 cells *in vitro*, which encouraged us to develop new [^18^F] labeled DPAZn2 compounds for apoptosis PET imaging *in vivo*. [^18^F]FP-DPAZn2 radiolabeled with [^18^F] was successfully prepared by a two-step labeling procedure with a total synthesis time of 110 min. Time course biodistribution studies revealed organ-specific variations in [^18^F]FP-DPAZn2 retention and pharmacokinetics, characterized by an initial uptake in the kidney and liver (at 5 min post-injection, 20.99% and 10.97% ID/g) and clearance primarily through the urinary tract (at 60 min post-injection, 9.30% ID/g) and, to a lesser extent, hepatobiliary (at 60 min post-injection, 4.01% ID/g) elimination. After 2 hours, several organs, including the kidney, the intestine, the pancreas and the liver, had a relatively high uptake (>2% ID/g), possibly because of the hydrophobicity of the tracer. An important advantage of this rapid excretion rate is that the background levels of uptake in nonapoptotic tissues are lower than with the [^18^F]-labeled protein. We further clarified the nature of the radioactive species in the cardiovascular system. Time course biodistribution studies revealed that the accumulation of [^18^F]FP-DPAZn2 radioactivity in the myocardial tissue was low and decreased from 4.4 to 1.4 % ID/g. PET imaging also showed a very low uptake of [^18^F]FP-DPAZn2 in normal hearts from 30 to 60 min post-injection.

The rat model of AMI is considered to be a good, reproducible, and cost-effective system for testing apoptosis. After an infarction, the process of ventricular remodeling begins rapidly, usually within a few hours, and it continues to develop for almost a week [22]. In the present study, AMI rats were confirmed by a larger LVEDD, a thinner IVS and a lower LVEF than normal rats using echocardiography. [^18^F]FP-DPAZn2 PET imaging showed that the myocardial uptake of [18F]FP-DPAZn2 was significantly increased in AMI rats compared with normal rats at 60 min post-injection. Similarly, a higher heart-to-muscle uptake ratio of [^18^F]FP-DPAZn2 was exhibited in AMI rats than in normal rats at 60 min post-injection. Of note, the study showed that [18F]FP-DPAZn2 accumulated much more in the hearts of normal rats at 15 and 30 min post-injection. Additionally, the bio-distribution study showed a high heart-to-muscle uptake ratio in normal rats at 15 min post-injection. We speculate that the relatively high overall plasma concentration (6.3-2.8 % ID/g over 5-30 min) and plasma half-life (22.5 min) of [^18^F]FP-DPAZn2 may account for the increased normal tissue retention. In addition, the great variability among animals could not be excluded.

[^18^F]FP-DPAZn2 PET imaging was confirmed by the TUNEL assays of cardiac tissues and histologic examination *in vitro*. The uptake of [^18^F]FP-DPAZn2 was strongly correlated with results of an independent histological analysis of AMI rats. Furthermore, a high background uptake of [^18^F]FP-DPAZn2 in PET images was seen in the liver and kidneys, which was consistent with the biodistribution of [^18^F]FP-DPAZn2 in normal mice. Additionally, the activity of [^18^F]FP-DPAZn2 was rapidly cleared from the liver and kidneys within 90 min, and this clearance rate was more favorable than that of ^18^F-annexin V, which slowly cleared through the liver and kidneys within 2 h post-injection [13–15]. We compared heart [^18^F]FP-DPAZn2 uptake with ^18^F-FDG uptake and retention. Although ^18^F-FDG is used for detecting myocardial viability, it is presently the gold standard for myocardial imaging in clinical settings. A comparison between the time-integrated activity coefficients of the two radiotracers in the heart showed that the [^18^F]FP-DPAZn2 radiotracer retention was lower in the heart than that of 18F-FDG, indicating the limitation of [^18^F]FP-DPAZn2 in clinical applications. The other limitation of [^18^F]FP-DPAZn2 is that it does not appear to provide an advantage over ^18^F-FDG for high background uptakes in normal tissues and blood. All of the results indicated that [^18^F]FP-DPAZn2 is suitable for the *in vivo* PET imaging of cardiomyocyte apoptosis, but the low heart uptake of [^18^F]FP-DPAZn2 and its high background uptake limit its use for further clinical experiments.

Thus, [^18^F]FP-DPAZn2 uptake should ideally be considered as a measure of myocardial apoptosis. The potential value of [^18^F]FP-DPAZn2 was investigated to assess cardiomyocyte apoptosis *in vivo* PET imaging. However, it will be necessary to optimize its structure for improving myocardial uptake as a PET imaging tracer.

**Figure 6:**
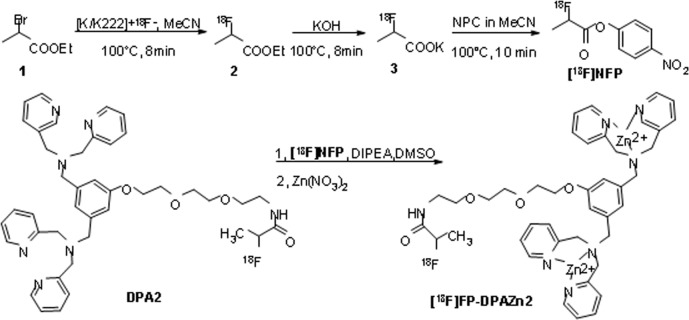
Radiosynthesis of [^18^F]FP-DPAZn2 from [^18^F]NFP

## CONCLUSIONS

In summary, a novel DPA probe, [^18^F]FP-DPAZn2, capable of imaging apoptosis, was synthesized. PET imaging and histological analysis suggested that [^18^F]FP-DPAZn2 is an effective tracer for myocardial cell apoptosis both *in vitro* and *in vivo*. Further studies are expected to optimize its structure for improving its myocardial uptake.

## MATERIALS AND METHODS

### Chemical synthesis and radiosynthesis

All of the chemicals obtained commercially were of analytical grade (Sigma-Aldrich, Milwaukee, WI, USA) and were used without further purification. Sep-Pak light QMA, Sep-Pak plus C18 and Oasis HLB cartridges were obtained from Waters Corporation (Milford, MA, USA). Sep-Pak light QMA cartridges were pre-conditioned with aqueous NaHCO_3_ (8.4 %) and water before use. Sep-Pak plus C18 and Oasis HLB cartridges were preconditioned with ethanol and water before use.

### Synthesis of dansyl-DPAZn2

The precursor DPA2 was prepared according to a procedure previously described in the literature [27] with slight modifications. After DPA2 (300 mg, 0.46 mmol) was dissolved in 15 mL of acetonitrile, 3 mL of triethylamine (56.7 mg, 0.56 mmol) and 20 mL of dry dichloromethane were added, which were cooled to 0°C in an ice bath, and then dansyl chloride (149 mg, 0.56 mmol) was added. The mixture was stirred for 3 h at room temperature under the protection of nitrogen and argon. The solvent was evaporated, and 20 mL of water was added to the reaction system. The mixture was extracted with ethyl acetate (4×20 mL), and the collected ethyl acetate layer was dried over anhydrous Na_2_SO_4_. Dansyl-DPA2 was purified with flash chromatography (ammonia methanol solution: ethyl acetate = 1:15) as a green oil (0.32 g, 79%). Dansyl-DPAZn2 was prepared from dansyl-DPA2, which was dissolved in trace ethanol. Two chemical equivalents of Zn(NO_3_)_2_ (aqueous solution, 15 mM) were added, and the mixture was heated at 70°C for 10 min and used without further purification. The desired product, dansyl-DPAZn2, was confirmed with 1H NMR (CDCl3, 400 MHz) (ppm): 8.51 (d, 5H, J = 4Hz), 8.30(d,1H), 8.24(d,1H),7.60-7.48(m, 8H), 7.48(t, 2H), 7.06-7.14(m, 4H), 7.07(s, 1H), 6.89(s, 2H), 4.14-4.13(m, 2H), 3.78(s, 10H), 3.67 (s, 2H), 3.55-3.54(m, 2H), 3.39(m, 4H), 3.11(d, 2H), 2.89(s, 6H), 2.04 (s, 1H), 2.00(s,1H) [18].

### Radiosynthesis of [^18^F]FP- DPAZn2

[^18^F]FP-DPAZn2 was synthesized from [^18^F]NFP as shown in Figure 6. [^18^F]NFP, a versatile ^18^F-labeling prosthetic reagent for radiolabeling peptides and proteins, was prepared from bis(4-nitrophenyl) carbonate (NPC) with the PET-MF-2 V-IT-I synthesizer as previously reported [19, 28]. After this product was obtained by collecting the corresponding fraction from semi-preparative HPLC and concentrated by a C18 cartridge, the purified [^18^F]NFP was eluted with ether and further dried with an anhydrous sodium sulfate (Na_2_SO_4_) cartridge. The [^18^F]NFP ether solution was evaporated to dryness under a stream of nitrogen at room temperature. A solution of DPA2 (100 μg) in 20 μL of DIPEA and 0.2 mL of anhydrous DMSO was added to the dried [^18^F]NFP residue. The reaction mixture was kept at room temperature for 5 min, and then it was quenched with 0.5% acetic acid (1 mL) and water (8 mL). The mixture solution was passed through a Sep Pak plus C18 cartridge (Waters, USA), and the cartridge was washed with water (10-15 mL). The desired product [^18^F]FP-DPA2 was eluted from the cartridge with 2 mL of ethanol. Zn(NO_3_)_2_ (15 mM aqueous solution, 10μl) was added to the solution of [^18^F]FP-DPA2 in ethanol, and the solution was heated at 70°C for 10 min. Finally, the solution of ^18^F-FP-DPAZn2 was formulated in normal saline and passed through a 0.22 μm Millipore filter into a sterile vial for the next experiments.

### Cell lines and cell culture

The H9C2 *cardiomyocyte* cell line was purchased from the Cell Bank of the Shanghai Institute of Biochemistry and Cell Biology of the Chinese Academy of Sciences. The cells were maintained in Dulbecco's Modified Eagle's Medium (DMEM) with 10% fetal bovine serum, 100 IU/ml penicillin, and 100 μg/ml streptomycin at 37°C in 5% CO_2_.

### Western blotting

Cell lysates were separated by SDS-polyacrylamide gel electrophoresis and transferred onto nitrocellulose membranes. The membranes were incubated with primary antibody overnight at 4°C. The following primary antibodies specific to the following proteins were used: β-actin (1:10000, Sigma, US), Bcl-2 (1:500, CST, US) and Bax (1:500, CST, US). The membranes were probed with HRP-conjugated secondary antibodies. The immunoreactive blots were visualized using an enhanced chemiluminescence reagent (Pierce, Rockford, IL, USA).

### Terminal deoxynucleotidyl transferase-mediated dUTP nick end labeling (TUNEL)

For assessing apoptosis, H9C2 cells in 24-well plates (1×10^5^ cells/well) were treated with doxorubicin (Dox 0.2 mg/l) in growth medium for 48 h at 37°C. The TUNEL assay was performed according to a standard protocol. For the TUNEL assay, an *in situ* cell death detection kit (Boster Biological Technology, China) was used according to the manufacturer's instructions. Briefly, the sections and coated H9C2 cells were treated with proteinase K (20 μg/ml) and incubated with the reaction mixture containing TdT and fluorescein-labeled dUTP for 1 h at 37°C. Images were *captured with* a microscope (Olympus, Japan) equipped *with a* CCD camera. For a negative control, TdT was omitted from the reaction mixture.

### Immunofluorescent confocal imaging

Fluorescein isothiocyanate-annexin V (FITC-annexin V) and propidium iodide (PI) assay kits were purchased from Biovision (BD, CA, USA). Doxorubicin (Dox) was obtained from Sigma. To compare the dansyl-DPAZn_2_ probe with FITC-annexin V for staining cells, H9C2 cells in 24-well plates (1×10^5^ cells/well) were treated with Dox (0.2 mg/l) in growth medium for 48 h at 37°C. The cells were washed twice with ice cold phosphate buffer solution (PBS) and triply stained with dansyl-DPAZn2 (50 μg/ml), FITC-annexin V and PI for 15 min at 37°C. The staining pattern was washed and evaluated under an Olympus fluorescence microscope. For PI staining, excitation at 488 nm and emission at 617 nm (band pass) were used, whereas for FITC detection, the excitation was at 488 nm and the emission was at 519 nm. The corresponding filters with excitation at 320-350 nm and emission at 420-460 nm were used for the detection of DPA-Zn2.

### Animal models

The animal experiments were approved by the Shanghai Ninth People's Hospital Animal Care and Ethics Committee. All of the experiments were conducted in accordance with the Guide for the Care and Use of Laboratory Animals published by Shanghai Ninth People's Hospital Affiliated to *Shan*ghai JiaoTong University School of Medicine.

Male Sprague-Dawley rats (*n* = 10) weighing 200 to 220 g were anesthetized by an intraperitoneal administration of 2% pentobarbital (40 mg/kg). The rats were endotracheally intubated and mechanically ventilated (JT3TKR-200C, Beijing, China) with supplemental oxygen. The heart was exposed through a thoracotomy at the left fourth intercostal space, and the left anterior descending coronary artery (LAD) was occluded by a 6-0 silk suture 1 to 2 mm below the tip of the left atrial appendage. The presence of an acute myocardial infarction (AMI) was confirmed by the pale appearance of the area at risk and changes in the electrocardiography profiles, including an immediate elevation of the ST segment and a significant increase in the amplitude and width of the QRS complex. After obstruction, the lungs were fully inflated by positive end-expiratory pressure. The chest cavity, muscles, and skin were sutured in 3 layers. Sham animals (*n* = 8) were submitted to the same protocol except for ligating LAD.

### *In vivo* biodistribution

For the ^18^F-FP-DPAZn2 biodistribution studies, rats were anesthetized with 2% pentobarbital (40 mg/kg) before injecting the radiotracer. The rats were injected with ∼1.48 MBq (∼40 μCi) of ^18^F-FP-DPAZn2 in 100−200 μL of saline through the tail vein. The radioactivity in the syringe before and after administration was measured in a calibrated ion chamber. A prescribed duration of time, 5, 30, 60 and 120 min post-injection, was allowed before procuring the organs and tissues. Blood was obtained through the ophthalmic vein, and other tissue samples of interest, including heart, brain, muscle, liver, lung, kidney, stomach and small-intestine tissues, were rapidly dissected and weighed. The ^18^F radioactivity was measured with an autogamma counter. All of the measurements were background-subtracted and decay-corrected to the time of injection then averaged together. The tissue radioactivity was expressed as the percentage of injected dose per gram of tissue (% ID/g).

### Echocardiography

To follow the time-course of the left ventricle (LV) function and remodeling after AMI, at one day after inducing AMI, echocardiography was performed using the German Acuson Sequoia C512 ultrasound heartbeat graph examination instrument equipped with a linear 8-14 MHz transducer. The LV dimensions were obtained from a long-axis view by two-dimensional guided M-mode imaging. The left ventricle end-systolic (LVESD) and end-diastolic (LVEDD) dimensions, as well as the interventricular septum (IVS) and the LV posterior wall thickness (LVPW), were measured from the M-mode tracings. The LV ejection fraction (LVEF) was calculated from the M-mode LV dimensions using the following equations: LVEDV = 7×LVEDD^3^/(2.4+LVEDD); LVESV = 7×LVESD^3^/(2.4+LVESD); LVEF = (LVEDV-LVESV)/LVEDV×100%.

### PET imaging

On one day after AMI induction, the animals were scanned on the Inveon small-animal PET/computed tomography (CT) scanner (Siemens) in three-dimensional acquisition mode. After the intravenous injection of [^18^F]FP-DPAZn2 (2.4∼3.7 MBq, 300∼400 μCi/Kg), the rats were anesthetized via the intraperitoneal injection of 2% pentobarbital (40 mg/kg), and PET images were obtained at four time points (30, 60, 90 and 120 min) post-injection. The CT scan was used for the attenuation correction and localization of the lesion site. Images were reconstructed by the two-dimensional ordered-subsets expectation maximum (OSEM). For the small-animal PET scan, regions of interest (ROIs) were drawn over the heart on the decay-corrected whole-body coronal images using the Inveon Research Workplace 4.1 software. The radioactivity concentration (i.e., accumulation) in the heart was obtained from the mean pixel value within the multiple ROI volume, which was converted to MBq/mL using a conversion factor. Assuming the density of tissue was 1 g/cm^3^, the ROIs were converted to MBq/g and then divided by the administered activity to obtain an imaging ROI-derived % ID/g [29]. After PET-CT imaging, the blood was collected, the tissues of the heart were excised and weighted, and the radioactivities of these tissues were measured with the gamma counter.

### Histological analysis

After the PET imaging was complete, all of the surviving rats were killed, and their hearts were excised, washed with ice-cold saline, blotted dry and weighed. The tissue samples were fixed in 10% buffered formalin, embedded in paraffin, and cut into 4-μm-thick sections that were stained with hematoxylin-eosin.

### Statistical analysis

All of the data are expressed as the means + SD. Differences among the various groups were tested for statistical significance using Student's t test (Prismv5.0 software, GraphPad). A P value < 0.05 was considered statistically significant.
